# Inhibitory Mechanism of IL-6 Production by Orento in Oral Squamous Cell Carcinoma Cell Line CAL27 Stimulated by Pathogen-Associated Molecular Patterns from Periodontopathogenic *Porphyromonas gingivalis*

**DOI:** 10.3390/ijms24010697

**Published:** 2022-12-31

**Authors:** Yasuhiro Imamura, Yoshimasa Makita, Kazuya Masuno, Hourei Oh

**Affiliations:** 1 Department of Pharmacology, Matsumoto Dental University, Nagano 399-0781, Japan; 2 Department of Chemistry, Osaka Dental University, Osaka 573-1121, Japan; 3 Center of Innovation in Dental Education, Osaka Dental University, Osaka 573-1121, Japan

**Keywords:** orento, Kampo medicine, periodontal disease, oral cancer, toll-like receptor

## Abstract

Orento is a traditional Japanese medicinal kampo preparation that is also prescribed in oral care. In oral squamous cell carcinoma cell line CAL27, orento significantly inhibited periodontopathogenic bacterium *Porphyromonas gingivalis* lipopolysaccharide (LPS) and lipoproteins (PAMP)-stimulated production of interleukin (IL)-6. This suggests that orento negatively regulates PAMP-mediated toll-like receptor (TLR) signaling. Orento significantly suppressed PAMP-stimulated activation of the IL-6 promoter, indicating that orento may suppress the production of IL-6 by PAMP at the transcriptional level. Orento also suppressed TLR-mediated activation of transcription factor nuclear factor-kappa B (NF-kB) that was stimulated by PAMP. This finding indicates that orento may suppress the function and activation of factors involved in TLR signaling, thereby suppressing NF-kB-dependent expression of various genes. Orento suppressed IL-1 receptor-associated kinase (IRAK4), IRAK1, and c-Jun N-terminal kinase (JNK) phosphorylation in PAMP-stimulated CAL27 cells. This result indicates that orento is involved in the initiation of TLR signaling by PAMP and suppresses the downstream signaling pathways of myeloid differentiation primary response gene 88 (MyD88) such as mitogen-activated protein kinase (MAPK) and NF-kB cascades. These findings suggest that orento has an inhibitory effect on the production of inflammatory cytokines.

## 1. Introduction

In Japanese medicine, Kampo medicine is sometimes used as a pharmacotherapy option in addition to Western medicines. Kampo medicine is one of the traditional herbal medicines, which was originally developed in Japan from Chinese medicine [1]. Currently, there are 148 types of Kampo medicine preparations in Japan, and physicians use different types depending on the disease and symptoms. Orento is one of the Kampo medicine and is composed of seven components, namely coptis rhizome, processed ginger, cinnamon bark, pinellia tuber, ginseng, glycyrrhiza, and jujube. Orento is mainly used to treat symptoms such as the feeling of stagnation and disturbance of the stomach, and loss of appetite [1]. In clinical practice, orento is indicated for the treatment of acute gastritis, hangover, stomatitis, and periodontitis [2]. A recent clinical report indicated that orento was effective in treating oral lichen planus [3].

Recently, epidemiological studies of the risk of cancer in patients with periodontal disease have been reported. Cytokines produced at the site of periodontitis have been implicated in the risk of cancer in patients with periodontal disease [4,5]. Periodontal disease is a chronic disease that persistently irritates oral tissues. *gingivalis* is an anaerobic Gram-negative bacterium that is a typically involved in periodontal disease [6]. *P. gingivalis* can adhere to and invade host cells [7], and is significantly more abundant in gingival tissues of oral squamous cell carcinoma than in normal mucosa [8]. Orento is reported to have anti-inflammatory effects on gingival cells and osteoblasts [9]. However, these studies only confirmed the effect of IL-6 and IL-8 on suppressing inflammatory cytokines, the mechanism of action of orento at the molecular level has not been elucidated so far.

Several circulating and cell membrane-associated proteins have been shown to collaborate with TLRs in sensing microbial ligands and promoting inflammatory responses. [10]. IL-6 is a typical inflammatory cytokine that affects not only inflammation but also the biological activity of cancer cells [11]. The purpose of this study was to examine the effects of orento on TLR-mediated IL-6 production by PAMP stimulation in oral squamous cell carcinoma.

## 2. Results

### 2.1. Effects of Orento on Cell Survival

**Figure 1:**
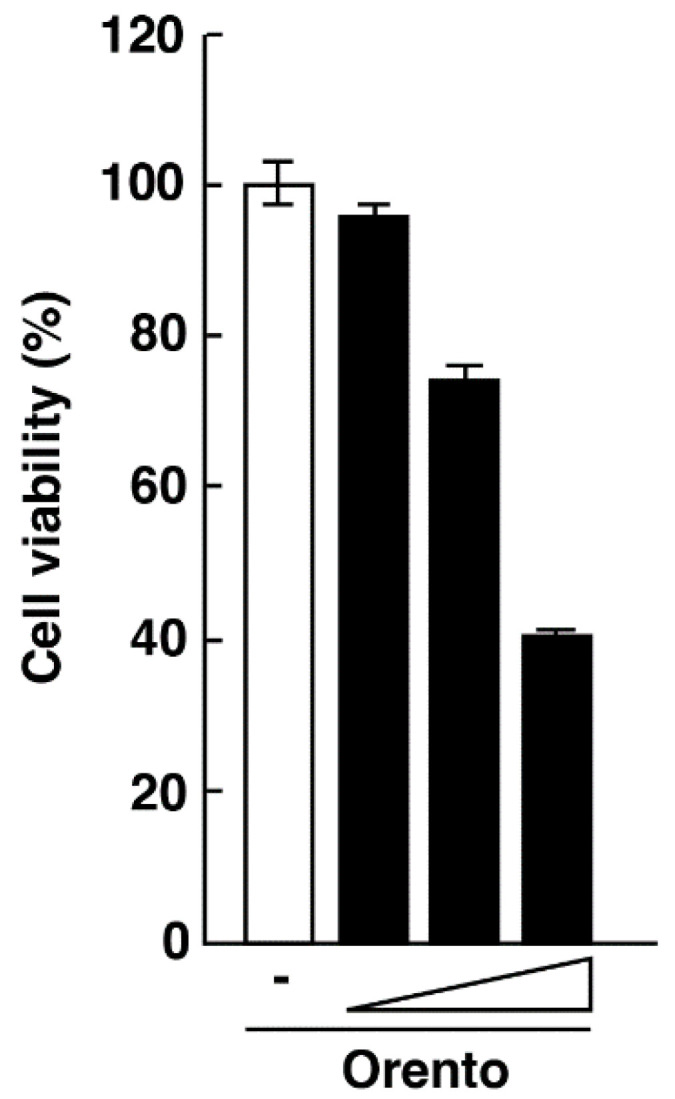
Effect of orento on survival of CAL27 cells. CAL27 cells were cultured with orento (10, 100, and 1000 μg/mL) for 1 day and MTT assay was performed. Bars represent the means and range of triplicate samples.

In order to elucidate the anti-inflammatory mechanism of action of orento, the effect of orento on the survival of CAL27 cells was first examined. CAL27 cells were cultured with orento (10, 100, and 1000 μg/mL) for 1 day and MTT assay was performed. As can be seen in Figure 1, orento decreased the viability of CAL27 cells at concentrations of 100 and 1000 μg/mL in a dose-dependent manner but had no effect at 10 μg/mL. Thus, 10 μg/mL was the concentration used in the subsequent experiments in this study.

### 2.2. Effects of Orento on PAMP-Stimulated IL-6 Production and Activation of Its Promoter in CAL27 Cells

**Figure 2:**
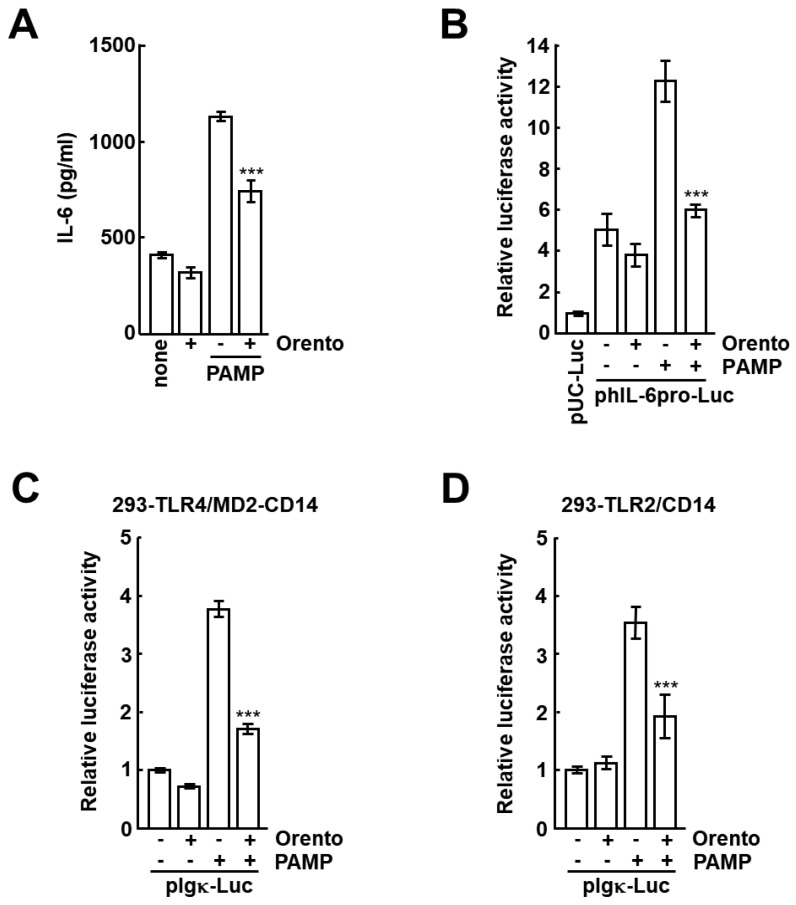
The effects of orento on increase in IL-6 production and the activation of its promoter by PAMP in CAL27 cells. The protocols are described in Section 4. (**A**) Orento and PAMP were added to the culture media of CAL27 cells, and the levels of IL-6 in the culture media were measured by ELISA. (**B**) A reporter plasmid containing the IL-6 promoter linked to the luciferase gene (phIL-6pro-Luc) and a standard plasmid (pRSV-β-gal) were co-transfected into CAL27 cells. (**C**,**D**) Inhibitory effects of orento on PAMP-stimulated NF-kB-dependent transcriptional activation. pIgκ-Luc and pRSV-β-gal were co-transfected into 293-TLR4/MD2-CD14 and 293-TLR2/CD14 cells. The cells were stimulated with PAMP (100 ng/mL) for 6 h in the presence of orento (1000 μg/mL). Luciferase and β-galactosidase assays were performed. The transcriptional activities were indicated as fold values of those of pUC-Luc (a plasmid containing luciferase gene alone) (**B**) and of pIgκ-Luc (without orento and PAMP stimulation) (**C**). Bars represent the means and range of triplicate samples. ***, *p* < 0.001 versus stimulation with PAMP without orento.

We examined the effect of orento on IL-6 production stimulated by PAMP. Orento and PAMP were added to the culture media of CAL27 cells, and the levels of IL-6 in the culture media were measured by ELISA. As can be seen in Figure 2A, PAMP-stimulated IL-6 production in CAL27 cells in the presence of orento, as compared to that in cells stimulated by PAMP alone, significantly decreased (*p* < 0.001). The level of IL-6 production in the presence of orento alone was similar to that in the absence of any stimulation. These results suggest that orento reduces IL-6 production by PAMP stimulation in CAL27 cells. Because IL-6 production may be regulated at the transcriptional level, we examined whether orento affects the transcriptional activation of the IL-6 promoter in CAL27 cells. A reporter plasmid containing the IL-6 promoter linked to the luciferase gene was transfected into CAL27 cells. The cells were stimulated with PAMP in the presence of orento, and luciferase assay was performed. As can be seen in Figure 2B, PAMP induced promoter activation, but orento alone did not. When orento was added to the culture medium of CAL27 cells, the increase in promoter activity triggered by PAMP was significantly reversed. These results suggest that orento represses the transcriptional activation of IL-6 promoter by PAMP stimulation. Next, we examined the effect of orento on NF-kB activation by reconfirming whether the PAMP used in this study has activity against TLR4 and TLR2. An NF-kB-dependent luciferase reporter plasmid was transfected into the stable cell lines 293-TLR4/MD2-CD14 (HEK293 cells constitutively expressing TLR4, MD2, and CD14) and 293-TLR2/CD14 (HEK293 cells constitutively expressing TLR2 and CD14). The cells were stimulated by PAMP in the presence of orento, and luciferase assay was performed. As can be seen in Figure 2C,D, the level of NF-kB-dependent transcriptional activation by PAMP stimulation was approximately 3.5- to 3.8-fold higher than that in the absence of any stimulation, but was significantly decreased by orento stimulation. The NF-kB activation in the presence of orento alone was not observed. These results suggest that orento inhibits the TLR-mediated NF-kB-dependent transcriptional activation by *P. gingivalis* PAMP.

### 2.3. Effect of Orento on Protein Phosphorylation involved in PAMP-Mediated Signal Transduction Cascades

**Figure 3:**
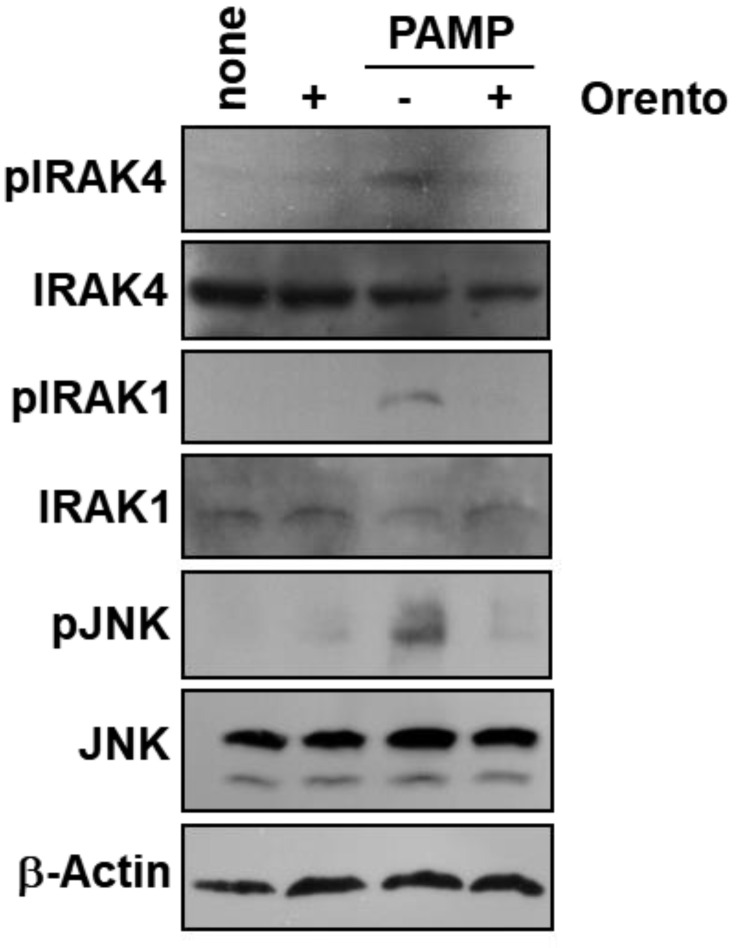
The effects of orento on PAMP-induced protein phosphorylation in CAL27 cells. CAL27 cells were cultured with 10 μg/mL orento for 24 h and stimulated with 100 ng/mL PAMP for 30 min. The cells were lysed and Western blotting was performed using antibodies against the proteins indicated in the figure.

In this study, we demonstrated the reduction of PAMP-stimulated IL-6 production by orento (Figure 2A). Orento was also found to inhibit PAMP-stimulated NF-kB-dependent transcriptional activation (Figure 2) Thus, we examined whether orento affects the activation (phosphorylation) of molecules in TLR-mediated signal transduction pathways. CAL27 cells were stimulated with PAMP in the presence or absence of orento, and the extracted proteins were analyzed by Western blotting. As can be seen in Figure 3, enhanced phosphorylation of IRAK4, IRAK1, and JNK was observed in response to PAMP stimulation, whereas orento dramatically decreased this effect. These results suggest that orento inhibits IRAK4 phosphorylation by acting at an earlier stage of PAMP-induced TLR activation.

### 2.4. Inhibitory Effect of Orento on Binding between MyD88 and IRAK4

**Figure 4:**
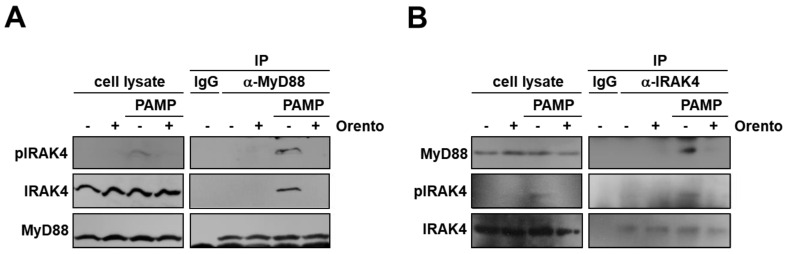
The effects of orento on binding between MyD88 and IRAK4 under PAMP stimulation in CAL27 cells. (**A**) Proteins in the cell lysates described in the legend of Figure 3 were immunoprecipitated (IP) using anti-MyD88 antibody, with IgG being the control. Precipitates and cell lysates were analyzed by Western blotting using anti-phosphorylated IRAK4 (top), anti-IRAK4 (center), and anti-MyD88 (bottom) antibodies. (**B**) Proteins in the cell lysates were immunoprecipitated using anti-IRAK4 antibody, with IgG being the control. Precipitates were analyzed using Western blotting using anti-MyD88 (top), anti-phosphorylated IRAK4 (center), and anti-IRAK4 (bottom) antibodies.

Because orento decreased the level of PAMP-stimulated IRAK4 phosphorylation (Figure 3), we next examined whether orento affects the binding of MyD88 and IRAK4. CAL27 cells were stimulated with PAMP in the presence or absence of orento, and MyD88 in the cell lysate was immunoprecipitated using anti-MyD88 antibody. Then, Western blotting was performed using anti-phosphorylated IRAK4 and anti-IRAK4 antibodies. As can be seen in Figure 4A, phosphorylated IRAK4 was co-precipitated with MyD88 under PAMP stimulation alone, but not under that in the presence of orento. No precipitates of MyD88 and IRAK4 were observed when control IgG was used. Conversely, in the case of immunoprecipitation with anti-IRAK4 antibody followed by Western blotting with anti-MyD88 antibody, MyD88 was co-precipitated with phosphorylated IRAK4 under PAMP stimulation alone, but not under that in the presence of orento (Figure 4B). These results indicate that orento inhibits the PAMP-stimulated interaction between MyD88 and IRAK4 in CAL27 cells.

## 3. Discussion

In this study, we have shown for the first time that orento suppresses TLR signaling. Two major findings were elucidated in this study. Firstly, TLR signaling suggests that *P. gingivalis* PAMP stimulates CAL27 cells to produce the inflammatory cytokine IL-6. Secondly, the inhibition of PAMP-stimulated interaction between MyD88 and IRAK4 by orento may have anti-inflammatory effects in this experimental system. In fact, orento is recommended for the treatment of stomatitis patients [1]. 

Periodontal disease is a long-term chronic inflammation. *P. gingivalis* is an anaerobic periodontopathogenic Gram-negative bacterium that can attach to and invade host cells [6]. In gingival tissues of oral squamous cell carcinoma, *P. gingivalis* is detected significantly more frequently than in normal mucosa, and the detection rate is higher than that of *Streptococcus gordonii*, a noninvasive oral bacterium [8]. Furthermore, chronic and persistent periodontal disease has been reported to be a risk factor for preoral and oral cancer [12]. The relationship between periodontal disease and oral cancer has attracted attention because periodontopathic bacteria such as *P. gingivalis* have been found to be very abundant in oral cancer patients, including in their saliva [4]. Periodontal disease has also been linked to cardiovascular disease, premature birth, low birth weight, stroke, lung disease, and diabetes [13,14]. 

*P. gingivalis* PAMP used in the present study served as an agonist for TLR4 and TLR2 (Figure 2C,D). The results of experiments using TLR4 knockout mice have revealed that *P. gingivalis* LPS acts on TLR2 [15]. However, this is thought to be the effect of lipoproteins contaminating the LPS. Recently, the highly purified *P. gingivalis* LPS has been shown to be an agonist for TLR4. Thus, *P. gingivalis* PAMP used in this study might contain some agonists for TLR2 such as lipoproteins, and we examined the effects of orento on the signaling by the PAMP through TLR4 and TLR2, which are expressed in CAL27 cells [16]. This appears to reflect the fact that *P. gingivalis* LPS containing (bound) lipoproteins in the oral cavity can affect these TLRs.

TLRs activate signal transduction cascades after stimulation with PAMP. TLRs recruit adaptor proteins such as MyD88 [17]. MyD88-dependent signal transduction is initiated by the formation of myddosome, which includes MyD88 and the IRAK family [18]. IRAK1 is activated after autophosphorylation of IRAK4 [19]. Upon activation, IRAK1 recruits and activates TNF receptor-associated factor 6 (TRAF6), and TRAF6 signaling then triggers the MAPK (JNK and p38) pathway and activates NF-kB [20]. In this study, we showed that orento inhibits TLR signaling by blocking the PAMP-stimulated interaction between MyD88 and IRAK4, thereby inhibiting IL-6 production, which was found to be due to inhibition of IL-6 transcription by inactivation of its promoter (Figure 2A,B). Orento may therefore be useful for treating IL-6-mediated inflammation and for repressing expression of the inflammatory cytokine genes dependent on NF-kB activation [21].

Many of the drugs in traditional medicine are composed of several plant parts and extracts (coptis rhizome, processed ginger, cinnamon bark, pinellia tuber, ginseng, glycyrrhiza glabra, and Jujube) [2]. Traditional Chinese medicine has a long history of formulations being developed, as documented in classical Chinese books such as Shanghan Lun [1]. Orento consists of seven components, of which the main crude drug, oren, contains 5–10% of the alkaloid berberine [22] and is known for its bactericidal, bacteriostatic, antifebrile, anti-inflammatory, digestive organ analgesic, and central nervous system depressant effects [2]. These effects of oren, combined with the anti-inflammatory effects of saponin and glycyrrhizin in licorice, are thought to be effective against stomatitis.

The anti-inflammatory effects of berberine have been previously reported [22], and its active component has also been isolated. Recently, the anti-inflammatory effects of a herbal medicine of carica papaya leaf extract on interdental bleeding in healthy subjects have been reported [23]. Furthermore, it has been suggested that berberine exerts its anti-inflammatory effect by suppressing MAPK signaling and the production of reactive oxygen species. Berberine has been shown to suppress LPS-induced inflammatory cytokine production in macrophages [24]. Studies using animal models of diabetic nephropathy indicate that berberine inactivates NF-kB and suppresses kidney inflammation [25]. Based on our findings, the suppression of IL-6 production in this study was inferred to be caused by orento, and the previously demonstrated effect of berberine present in the oriental drug coptis rhizome supports the results of this study [9]. Orento, which contains coptis rhizome, inhibits phosphorylation (activation) of factors downstream of the signaling pathway by blocking the binding of MyD88 to IRAK4. As a result, NF-kB-dependent gene expression is suppressed. This inhibitory effect may be similar to the anti-inflammatory effects of berberine, which also contains coptis rhizome. 

In conclusion, orento suppresses IRAK4, IRAK1, and JNK phosphorylation in *P. gingivalis* PAMP-stimulated CAL27 cells. Orento is involved in the initiation of TLR signaling by PAMP and suppresses downstream signaling pathways such as MAPK cascade and NF-kB signaling activation by MyD88. Thus, orento may exert anti-inflammatory effects through TLR signal transduction.

## 4. Materials and Methods

### 4.1. Cell Culture

The cell lines, CAL27 (oral squamous cell carcinoma) (American Type Culture Collection, Manassas, VA, USA), 293-TLR4/MD2-CD14 (InvivoGen, San Diego, CA, USA), and 293-TLR2/CD14 (InvivoGen), were cultured in Dulbecco’s Modified Eagle’s Medium (DMEM) (Nissui pharmaceutical, Tokyo, Japan) with 10% fetal bovine serum (FBS), 100 units/mL penicillin G, and 100 μg/mL streptomycin, at 37 °C in a 5% CO_2_ and 95% air in a humidified incubator.

### 4.2. Reagents

**Figure 5:**
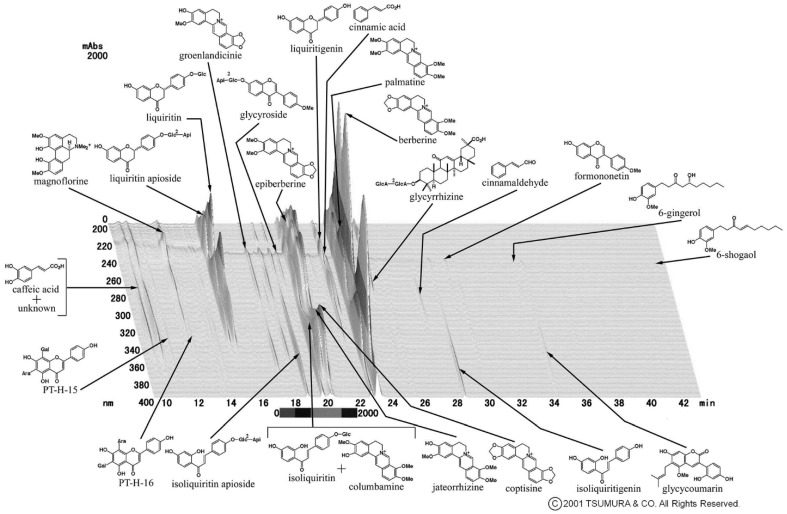
Three-dimensional high-performance liquid chromatography profile of orento (provided by Tsumura, Tokyo, Japan).

The following reagents and antibodies were purchased: orento (Tsumura, Tokyo, Japan); *P. gingivalis* LPS (InvivoGen, refer to “PAMP” in this study); 3-(4,5-dimethylthiazol-2-yl)-2,5-diphenyl tetrazolium bromide (MTT) (Sigma-Aldrich, St. Louis, MO, USA); Luciferase Cell Culture Lysis 5X x Reagent (Promega, Madison, WI, USA); streptavidin-horseradish peroxidase (HRP) (Thermo Fisher Scientific (Biosource), Waltham, MA, USA); SureBlue TMB Microwell Peroxidase Substrate (SeraCare Life Sciences, Milford, MA, USA); anti-IL-6, biotinylated anti-IL-6 antibodies (Thermo Fisher Scientific (eBioscience); anti-phosphorylated IRAK4 and anti-phosphorylated IRAK1 antibodies (Thermo Fisher Scientific (Invitrogen)); anti-IRAK4, anti-IRAK1, anti-phosphorylated JNK, anti-JNK, and anti-MyD88 antibodies (Santa Cruz Biotechnology, Dallas, TX, USA); anti-β-actin antibody (Abcam, Cambridge, UK). The chemical components of orento were analyzed by Tsumura, Tokyo, Japan using a three-dimensional high-performance liquid chromatography profile (Figure 5).

### 4.3. Plasmid Construction

The human IL-6 promoter region, which is the sequence spanning base pairs 2636 to 5035, with GenBank accession number NG_011640, was synthesized by Azenta Inc. (Chelmsford, MA, USA). This fragment was provided by cloning into pUC57-Kan (Azenta) (named pUC-Kan-hIL-6pro). The *Kpn* I-*Xho* I fragment of the IL-6 promoter from pUC-Kan-hIL-6pro was inserted into the same sites of pUC-Luc (in which the fragment containing the luciferase gene and SV40 polyadenylation signal from PGV-B2 (TOYO INK, Tokyo, Japan) was cloned into pUC18) (named phIL-6pro-Luc).

### 4.4. MTT Assay

CAL27 cells (1 × 10^4^) were cultured with 10, 100, and 1000 μg/mL of orento for 24 h. The subsequent procedures were performed as described elsewhere [26]. The cells were cultured with 10 mL of 5 mg/mL MTT for 4 h. After the addition of 0.04 N HCl-isopropanol (100 mL) to the medium, the produced formazan was completely dissolved. Then, samples were measured by the microplate reader (595/655 nm (test/reference)).

### 4.5. Enzyme-Linked Immunosorbent Assays (ELISAs)

The ELISAs were performed as described in the user manual of CytoSet kits (Thermo Fisher Scientific (BioSource)) [27]. CAL27 cells (1 × 10^4^) were cultured with a mixture of *P. gingivalis* PAMP (100 ng/mL) and orento (10 μg/mL) for 24 h. Then, the culture media were collected. IL-6 in the culture media was reacted with the anti-IL-6 (1 μg/mL) antibody for coating and the biotinylated anti-IL-6 (0.6 μg/mL) antibody for detection at 4 °C for 1 day. Then, streptavidin-HRP diluted 1000-fold was added to the above reaction solution and placed at r. t. for 1 h. After HRP had been reacted with peroxidase substrate at r. t. for 45 min, the samples were measured by the microplate reader (450/655 nm (test/reference)). 

### 4.6. Transfection and Luciferase Assay

phIL-6pro-Luc (1 μg) and pRSV-κ-gal (0.1 μg, standard plasmid) were mixed with TransIT-LT1 reagents (Mirus Bio, Madison, WI, USA). pIgκ-Luc (a reporter plasmid with the NF-kB binding sequence, 0.5 μg) and pRSV-κ-gal (0.05 μg) were also mixed with TransIT-LT1 reagents. After placed at r. t. for 30 min, the mixture containing phIL-6pro-Luc was added to CAL27 cells (3 × 10^5^). The mixture containing pIgκ-Luc was also added to 293-TLR4/MD2-CD14 and 293-TLR2/CD14 cells (3 × 10^5^). One day after transfection, the cells were incubated with orento (1000 μg/mL) for 24 h. The cells were then stimulated with *P. gingivalis* PAMP (100 ng/mL) and cultured for 6 h, followed by harvesting. The cells were lysed with luciferase cell culture lysis reagent at r. t. for 15 min. After centrifugation, the lysates were reacted with reaction solutions for luciferase and β-galactosidase and their activities were measured by the luminometer [27]. The experiments were performed in triplicate for each test group. The luciferase activities were normalized with the standard (β-galactosidase activity) and the values of various samples were compared to that of pUC-Luc, or of pIgk-Luc without stimulation. 

### 4.7. Western Blotting and Immunoprecipitation

Western blotting and immunoprecipitation analyses were performed as described previously [26,27]. Briefly, CAL27 cells (4 × 10^6^) were cultured in serum free DMEM containing 10 μg/mL orento for 24 h and then stimulated with 100 ng/mL *P. gingivalis* PAMP for 30 min. For Western blotting, the lysates from the above cells were prepared with RIPA buffer. After electrophoresis of the cell lysates, proteins were transferred to polyvinylidene difluoride membrane. Proteins on the membrane were reacted with the antibodies shown in Figure 3 and Figure 4. For immunoprecipitation analysis, MyD88 in proteins extracted from the above cells were reacted with anti-MyD88 antibody by rotating at 4 °C for 1 h. The protein–antibody complex was bound to protein G Sepharose at 4 °C for 1 h. The precipitates obtained by centrifugation were washed. After precipitates and cell lysates had been electrophoresed on 10% SDS-polyacrylamide gel, Western blotting was performed with anti-pIRAK4 and anti-IRAK4 antibodies. Conversely, immunoprecipitation with anti-IRAK4 antibody was followed by Western blotting with anti-MyD88 and anti-pIRAK4 antibodies. 

### 4.8. Statistical Analysis

Quantitative data were statistically analyzed using one-way analysis of variance (ANOVA) followed by Tukey’s test (StatMate software (ATMS, Chiba, Japan)). Differences were considered to be significant at *p* < 0.05.

## Data Availability

Not applicable.
